# Supplementary material for: Replication stress response in fission yeast differentially depends on maintaining proper levels of Srs2 helicase and Rrp1, Rrp2 DNA translocases
Source: PLoS One. 2024 Jun 21;19(6):e0300434. doi: 10.1371/journal.pone.0300434 (PMC11192394; doi:10.1371/journal.pone.0300434)
Supplement: S1 File — (PDF) [file pone.0300434.s001.pdf]

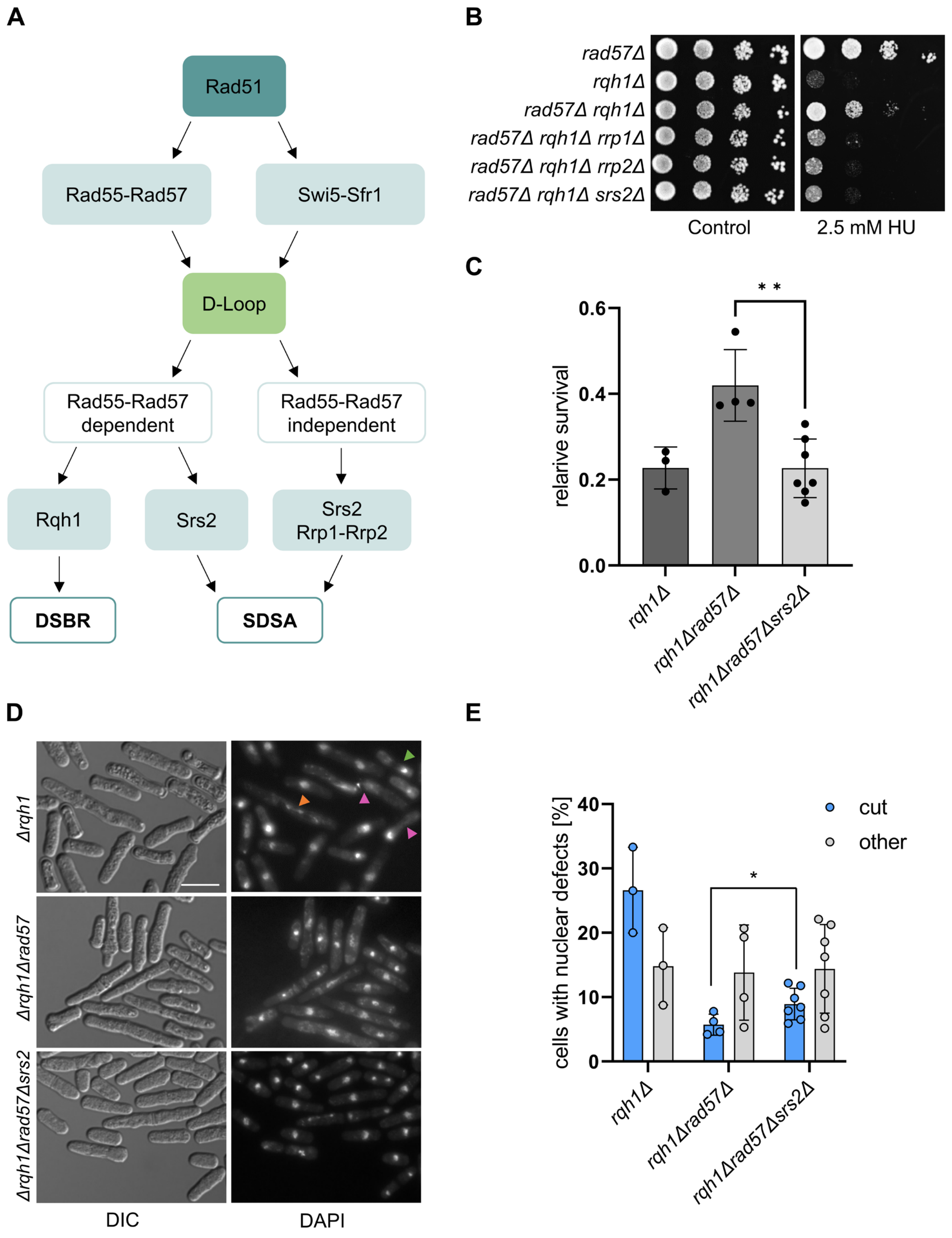
A
Rad51
Rad55-Rad57
Swi5-Sfr1
D-Loop
Rad55-Rad57 dependent
Rad55-Rad57 independent
Rqh1
Srs2
Srs2 Rrp1-Rrp2
DSBR
SDSA
B
rad57Δ
rqh1Δ
rad57Δ rqh1Δ
rad57Δ rqh1Δ rrp1Δ
rad57Δ rqh1Δ rrp2Δ
rad57Δ rqh1Δ srs2Δ
Control
2.5 mM HU
C
relarive survival
0.6
0.4
0.2
0.0
* *
rqh1Δ
rqh1Δrad57Δ
rqh1Δrad57Δsrs2Δ
D
Δrqh1
Δrqh1Δrad57
Δrqh1Δrad57Δsrs2
DIC
DAPI
E
cells with nuclear defects [%]
40
30
20
10
0
cut
other
*
rqh1Δ
rqh1Δrad57Δ
rqh1Δrad57Δsrs2Δ

Fig. S1

Fig S1. Srs2 helicase shares some functions in HR with Rrp1 and Rrp2 translocases. (A) A diagram of discussed Rad51 dependent homologous recombination pathways. Srs2 helicase is required for the rescue of *rqh1*Δ HU sensitivity (B) when cells were appropriately diluted, spotted on YES plates with 2.5 mM HU, incubated for 4 days and photographed; and HU induced viability loss (C) when after acute HU treatment (4 hrs in 12mM HU) cells of respective mutants and wild-type were diluted, plated on YES plates, incubated for 4 days. Resulting colonies were counted and relative survival of studied mutants with respect to wild-type strain was calculated. Values are means of at least three independent biological experiments. Error bars are standard deviation (SD). (D) The aberrant mitosis phenotype of the *rqh1*Δ mutant is attenuated by deletion of *rad57*+. Cells of studied mutants after 4 h recovery from 4 h treatment in 12mM HU were observed by DAPI staining. Cut nuclei are indicated by purple, lagging chromosomes by orange, and non-disjunction by green arrowheads. Scale bar represents 10 µm. (E) A graph presenting quantification of % of cut cells in respective mutants, with lagging and mis-segregated chromosomes grouped as “other”. At least three independent cultures of each strain were examined. Error bars represent the standard deviation about the mean values. Student’s t-test was performed to calculate P-values (* $0.01 < P \leq 0.05$).

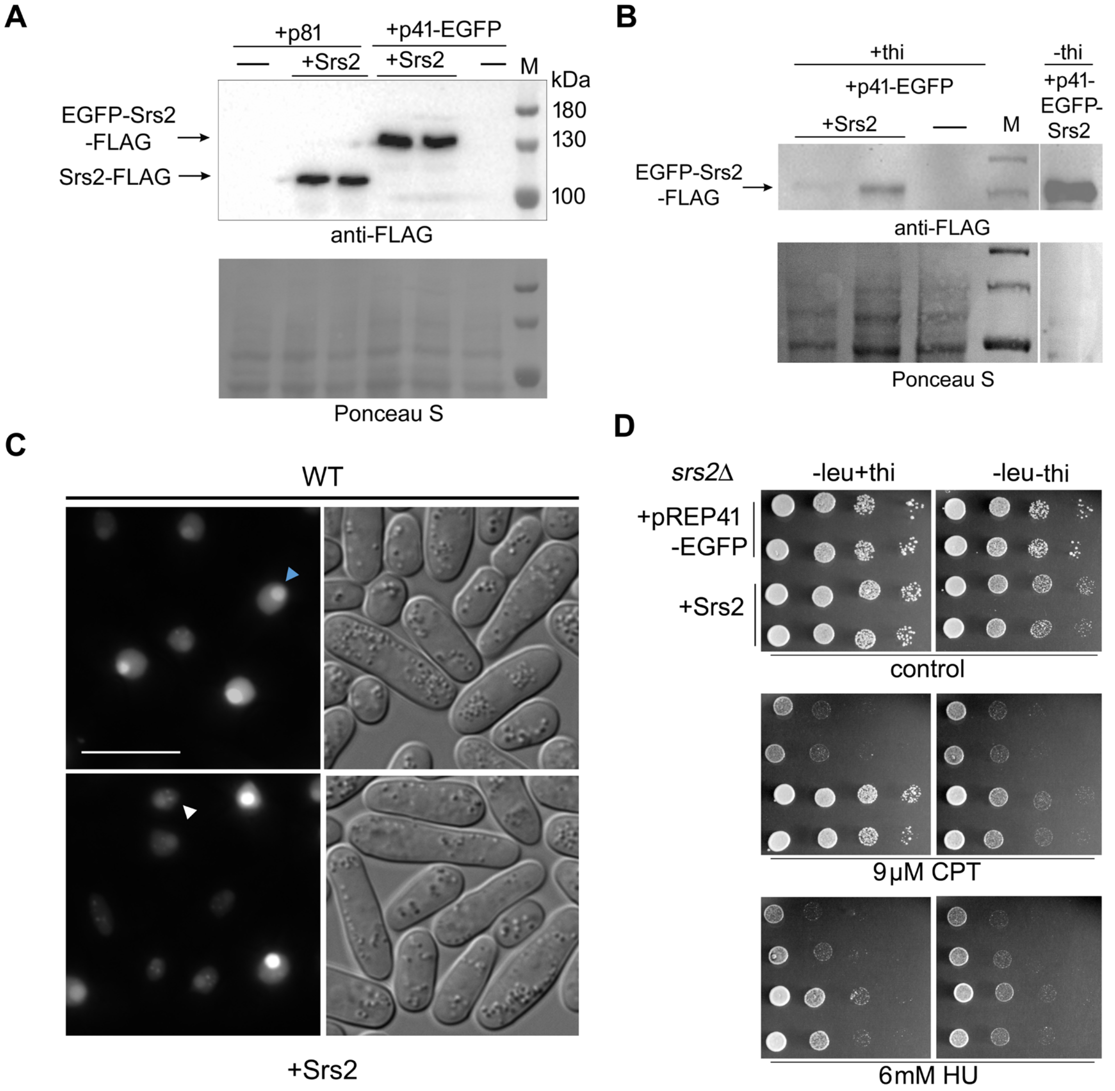

Fig. S2

Fig S2. Cloning of the *srs2*+ gene. (A) C-terminal FLAG-tagged *srs2*+ gene was cloned into several expression plasmids under medium (p41 or 42) or low (p81) strength *nmt* promoter. Western blot analysis with anti-FLAG antibody of protein extracts from transformants containing *srs2-FLAG* plasmids grown in minimal media without thiamine (under expressing inducing conditions, -thi). (B) Residual Srs2-FLAG protein is present even in cultures containing thiamine (+thi) when *nmt* promoter was repressed. (C) Overproduced EGFP-Srs2-FLAG localises to the nucleus in a form of diffused patches (blue arrowhead) and also forms spontaneous foci (white arrowhead) in wild type strain. Scale bar indicates 10 µm. (D) Transformation of *srs2*Δ strain with *EGFP-srs2-FLAG* expressing plasmid reversed its HU and CPT sensitivity. Cells were appropriately diluted and spotted on EMM plates with HU or CPT and supplemented or not with thiamine, incubated for 6 days and photographed.

S1 Table. Strains used in this study.

Strain	Genotype	Source
YA254 (WT)	ura4-D18, leu1-32, his3-D1, arg3-D1, h90	a
*rrp1*Δ	rrp1D:kanMX6, ura4-D18, leu1-32, his3-D1, arg3-D1, h90	b
*rrp2*Δ	rrp2D::kanMX6, ura4-D18, leu1-32, his3-D1, arg3-D1, h90	b
*rrp1*Δ	rrp1D::natMX6, his3-D1, leu1-32, ura4-D18, arg3-D1, h90	b
*rrp2*Δ	rrp2D::natMX6, his3-D1, leu1-32, ura4-D18, arg3-D1, h90	b
*srs2*Δ	srs2D::ura4+, ura4-D18, his3-D1, leu1-32, arg3-D4 h90	b
*rqh1*Δ	rqh1D::ura4+, ura4-D18, his3-D1, leu1-32, arg3-D4 h90	b
*rad57*Δ	rad57D::his3+, ura4-D18, leu1-32, his3-D1, arg3-D1, smt0	a
*rqh1*Δ*rhp57*Δ	rqh1D::ura4+, rhp57D::his3+, ura4-D18, his3-D1, leu1-32, arg3-D4 smt0	b
*rqh1*Δ*rhp57*Δ*rrp1*Δ	rqh1D::ura4+, rhp57D::his3+, rrp1D:*kan*MX6, ura4-D18, his3-D1, leu1-32, arg3-D4 smt0	b
*rqh1*Δ*rhp57*Δ*rrp2*Δ	rqh1D::ura4+, rhp57D::his3+, rrp2D:*kan*MX6, ura4-D18, his3-D1, leu1-32, arg3-D4, smt0	b
*rqh1*Δ*rhp57*Δ*srs2*Δ	srs2D::ura4+, rhp57::his3+, rqh1D:*kan*MX6 ura4-D18, his3-D1, leu1-32, arg3-D4, smt0	c
*rrp1*Δ*srs2*Δ	srs2D::ura4+, rrp1D::*kan*MX6, leu1-32, ura4-D18, his3-D1, h90	b
*rrp2*Δ*srs2*Δ	srs2D::ura4+, rrp2D::*kan*MX6, leu1-32, ura4-D18, his3-D1, arg3-D4, h90	b
*rad51*Δ	rhp51D::his3+, ura4-D18, his3-D1, leu1-32, arg3-D4 smt0	a
*rad3*Δ	rad3D::kanMX6, ura4-D18, leu1-32, ade6-704, h-	d
*cds1*Δ	cds1D::natMX6, ura4-D18, leu1-32, ade6-704, h-	d
*chk1*Δ	chk1D::kanMX6, ura4-D18, leu1-32, ade6-704, h-	d
*cds1*Δ*srs2*Δ	cds1D::natMX6, srs2D::ura4+, ura4-D18, leu1-32, ade6-704, his3-D1, arg3-D1, h-	c
*chk1*Δ*srs2*Δ	chk1D::kanMX6, srs2D::ura4+, ura4-D18, leu1-32, ade6-704, his3-D1, arg3-D1, h-	c
AW161	chk1-HA, ura4-D18, leu1-32, his3-D1, h+	d
CJ01	ura4-D18; leu1-32; his3-D1; arg3-D1; ade6-m210; Chr16 ade6-m216; h+	e
AMC377	rad11-GFP::kanMX6, ura4-D18, leu1-32, his3-D1, h+	d
AH 109 (*S. cerevisiae)*	MATa, trp1-901, leu2-3, 112, ura3-52, his3-200, gal4D, gal80D, LYS2::$GAL1_{UAS}$-$GAL1_{TATA}$-HIS3, $GAL2_{UAS}$-$GAL2_{TATA}$-ADE2, URA3::$MEL1_{UAS}$- $MEL1_{TATA}$-lacZ	Clontech
FY21311 (MY7291)	Leu1, ura4+, gar2+-mCherry:KanMX6, h-	NBRP, Japan

a - Hiroshi Iwasaki
b - laboratory stock

c - this work
d - Tony M Carr
e - Jo Murray

S2 Table. Plasmids used in this study.

Plasmid	Source
pREP81-FLAG	a
pREP81-Srs2-FLAG	b
pREP41-EGFP	c
pREP41-EGFP-Rrp1-FLAG	a
pREP41-EGFP-Rrp2-FLAG	a
pREP41-EGFP-Srs2-FLAG	b
pREP42-FLAG	c
pREP42-HA-Srs2-FLAG	b
pGADT7	d
pGBKT7	d
pGADT7-Rrp1	a
pGADT7-Rrp2	a
pGBKT7-Srs2	b

a - laboratory stock
b - this study
c - Craven,R.A., *et al.* (1998) Gene, 221, 59–68. doi:10.1016/s0378-1119(98)00434-x
d - Clontech

S3 Table. Primers used in this study.

Application/ Cloned gene	Primer name	Primer sequence
FLAG	REP3flag_a_r<br>REP3flag_b_f<br>REP3flag_c_r<br>REP3flag_d_f	ctttatcatcgtcgtccttgtagtcggatcctctagagtcgacatatgatttaac<br>tacaaggacgacgatgataaagactacaaggacgacgatgataaagacta<br>gggtcatttatcatcgtcgtccttgtagtctttatcatcgtcgtccttgtagt<br>caaggacgacgatgataaatgacccgggtaaaaggaatgtctcccttgccagtac
Srs2-FLAG	srsEx1_fwd<br>srsEx1_rev<br>srsEx2_fwd<br>srsEx2_rev<br>srsEx3_fwd<br>srsEx3_rev<br>srsEx4_fwd<br>srsEx4_rev	atagtcgctttgttaaatcatATGGAAACGAAATCATCATAC<br>aatagcttgaGTATCATTTCTGTCAGCAATC<br>gaaatgatacTCAAGCTATTATGAAGCGAC<br>catcaaaatcCGCTAAATTGTTCTTCCATAAG<br>caatttagcgGATTTTGATGATTTGCTTTTAAACTTTATTTTATTAC<br>gtttaatttctCTTGCGATCCAGTAAGATTC<br>ggatcgcaagAGAAATTAAACGTATAGTAGGTTC<br>catcgtcgtccttgtagtcggatccTAACATTCGTGAAACTCGTAG
ChIP-qPCR	rDNA_F	GGACGGTGGCCATGGAA
ChIP-qPCR	rDNA_D	CATTCGGCCGGTGAGTTG
ChIP-qPCR	Intergenic locus (II.50F)	CACCGCAGTTCTACGTATCCT
ChIP-qPCR	Intergenic locus (II.50R)	CGATGTAACGGTATGCGGTA
southern rDNA	KK146	GAATTCGGTAAGCGTTGGATTG
southern rDNA	KK147	GAATTCTTCTTTCACATCTCC
