# Supplementary material for: Replication stress response in fission yeast differentially depends on maintaining proper levels of Srs2 helicase and Rrp1, Rrp2 DNA translocases
Source: PLoS One. 2024 Jun 21;19(6):e0300434. doi: 10.1371/journal.pone.0300434 (PMC11192394; doi:10.1371/journal.pone.0300434)
Supplement: Attachment — Submitted filename: Response to Reviewers.docx [file pone.0300434.s002.docx]

**RESPONSE TO REVIEWERS' COMMENTS**

**General comments**

We would like to thank the Reviewers for their extensive and helpful comments. As suggested, we have performed additional experiments and rewritten parts of the text, and we hope the Reviewers will now find our manuscript satisfactorily improved.

Reviewer’s comments are in blue and our responses are in black font. Numbering of pages and figures refer to the revised version of the manuscript. All changes to the text of the manuscript are marked in yellow.

**Reviewer #1:** The study by Baranowska et al. genetically investigates the replication stress response in fission yeast and its dependence on maintaining proper levels of Srs2 helicase and Rrp1, Rrp2 DNA translocases. The upregulation of Srs2 helicase levels leads to enhanced replication stress, chromosome instability, and viability loss. Dysregulation of Srs2, Rrp1, and Rrp2 protein levels differentially affects checkpoint response. The study provides important insights into the regulation of replication stress and DNA repair processes in fission yeast.
Most data are of genetics, and sometimes it is hard to understand molecular relationship between factors, therefore authors should pay attention to illustrate molecular scheme underlying the genetic data, in text and in drawings.
Part of the conclusion has not been fully supported by the evidence provided in the submitted manuscript, which should be improved.
Specific comments are as listed below:

Introduction and Figure 1A,
Through introduction to Figure 1, It was not clear how Rqh1 is known to be involved in the Rad51-dependent HR events. Does Rqh1 work in the Rad55-Rad57 branch? In Figure 1A, rad57∆ is not sensitive to HU, but rqh1∆ is extremely sensitive, meaning that Rqh1 is involved in both Rad55 and SDSA pathways. Is this correct? It is required to explain the function of Rqh1 in the introduction and how we should interpret the spot assay showing intermediate sensitivity of rad57∆rqh1∆ against HU.

We agree with the Reviewer that it was difficult to follow the data presented in the Figure 1 without description of the HR pathways in which all studied proteins are involved. We have now prepared a diagram of the fate of D-loop formed by Rad51 with the help of two mediator complexes and marked positions where Rqh1, Srs2 and Rrp1/2 are thought to be involved. We placed it Supplementary Figure S1 together with the data introducing epistasis analysis between *rad57∆rqh1∆* with *rqh1∆* that refer directly to our previous analysis with *rrp1∆* and *rrp2∆* [1]. We hope that currently the figure is clear and constitutes a comprehensive starting point for data presented in this manuscript.

Figure 1B,
Evidence for the nuclei defects in photos (such as DAPI) is required.

We have now added, as Figure S1D, the representative photographs of DAPI stained cultures of 3 discussed mutants and indicated the examples of lagging, cut and mis-segregated chromosomes in the photograph depicting *rqh1*Δ mutant cells.

Figures 2C and 3:
The labels should be “+thi” and “-thi” on the top, as expression levels change depending upon thiamine in the medium. This is a common issue throughout the figures presented in the manuscript.

We changed the labelling of Figures throughout the manuscript, as requested.

Figure 2D,
Location of overexpressed EGFP-Srs2-FLAG needs to be clarified by use of a nucleolus marker at the same time with EGFP-Srs2.

We re-examined localisation of overexpressed EGFP-Srs2-FLAG in a strain expressing Gar2-mCherry, a nucleolar histone chaperone, and included these data in new Fig.3A. As before, we observed diffused localisation of Srs2 within nucleolus and also foci, often located outside nucleolus. We thus extended description of obtained data (lines 344-353). In order to confirm microscopy data, we performed ChIP experiment, now included as Fig. 3B, with description, line 351-353:

“Finally, we have confirmed by chromatin immunoprecipitation of EGFP-Srs2 followed by qPCR analysis that when overproduced Srs2 accumulates at DNA within rDNA region”.

p.7 l.204, “The double mutant of srs2 and rrp1 or rrp2 did not show any furhter delay,,, suggesting the role of Srs2 … may be independent of Rrp1 and Rrp2.”
I am a bit confused: the non-additive phenotype of srs2 rrp1/2 might indicate that those factors work in the same pathway rather than ‘independent’.

We are very grateful that this and other Reviewers pointed out this obvious mistake we made. We wrote precisely the opposite of what we meant to write. The whole point of overexpression analysis performed later was to examine if Srs2 has roles independent from Rrp1 and/or Rrp2. The relevant sentence now reads, line 255-258:

“The double mutants simultaneously devoid of *srs2+* and *rrp1+* or *rrp2+* did not show any further delay in the entry of chromosomes into the gel (Fig. 1B) suggesting that Srs2, Rrp1 and Rrp2 may have overlapping roles in the resolution of replication intermediates after HU block.”

P.8 L..249; Figure 3C, D,
For me toxicity of overexpressed Srs2 was not evident in rrp1∆ and rad51∆ cells, but was still visible in rrp2∆ cells. Clear images are to be presented if author’s statement is correct.

We substituted photographs of same plates after longer incubation, and we hope now the difference between the growth of *srs2*^+^ expressing cells and empty vector controls are more evident. We wish to point out that growth defect observed upon Srs2 overproduction, as well as Rrp1, is not very pronounced, and is often more readily observed on plates rather as smaller colonies than marked decrease in their number. We hope that when the Reviewer compares the effect of Srs2 overproduction in wild-type (Fig.2A) to that in respective mutants (Fig. 2D, E), they will agree that toxicity of Srs2 is the same in wild-type and *rrp1*Δ mutant and only slightly more pronounced in cells of *rrp2*Δ and *rad51*Δ mutants.

Figure 3F,
1) When yeast two-hybrid results is ‘negative’, we cannot conclude that the two factors do not interact. This applies particularly when there are no positive/negative controls for the factors. In Figure 3F, no positive controls for Rrp1 or Srs2 have been used, therefore the results might simply reflect that the two-hybrid system per se in Figure 3F does not work with those proteins. Authors need to take the possibility into account in results or in discussion.
2) co-IP experiments using S. pombe cells are to be performed.

We agree with the Reviewer that negative Y2H results do not prove lack of interaction between proteins, but we only argued in the text that direct interaction was probably absent.

We do not show controls with constructs used, but Y2H interaction between Rrp1 and Rrp2 as well as SUMO, was shown by us before using same constructs [2]. We have data on possible Srs2 self-interaction, pasted below, showing that this construct is also functional. We do not wish, however, to discuss this result in present manuscript. (Please note that for Rrp1 and Rrp2 on pGAD the growth on -3 medium results from self-activation due to the ability of Rrp1 and Rrp2 to bind to DNA.)

We performed co-IP experiments as suggested by the Reviewer. We obtained very preliminary result suggesting that EGFP-Srs2 may co-immunoprecipitate with Rrp1-HA (see red square in Western blot pasted below). Unfortunately, the signal was very weak and no reciprocal IP (Rrp1-HA with anti-GFP antibody) was observed. The result with Rrp2 was nonconclusive.

These are clearly technical issues and extensive optimisation of experimental conditions is required which we will certainly undertake. However, this will take time and would delay the publication of our findings. Therefore, we decided to emphasised this point in our description of these data, line 324-327:

“It is important to note, however, that such interaction may be mediated through other proteins, and needs to be investigated further. Nevertheless, the above data indicate that the effects of overexpression of these three genes may be, at least in part, independent of each other”.

We hope that the Reviewer agrees that for the purpose of this manuscript this experiment is not indespensible.

P.9 L.306 may affects > may affect

We corrected this mistake.

Figure 4C, D,
Images (such as DAPI) should be provided as evidence.

We included a representative photograph of wild-type cells overproducing Srs2-EGFP in Fig. 4C.

For chromosome assay, however, this is not practical. As stated in Materials and Methods, we counted the number of red among many white colonies on numerous YES low Ade plates, these events are not frequent. We do not have photographs of all those plates and hope that adding to figure a schematic diagram of the assay, together with the explanation, line 392-393:

”Cells that lose this mini-chromosome accumulate red intermediate due to the inability to complete adenine synthesis (Fig. 4E).”

will satisfy the Reviewer.

Figure 6,
As there are many factors appeared in the manuscript, and most data are of genetic analyses, a schematic summary may help understanding of readers.

We included a diagram of discussed HR pathways as Fig. 1SA and schematic summary of our conclusions as Fig. 7.

**Reviewer #2:** The authors’ group previously reported that DNA translocases Rrp1 and Rrp2 act together with the Srs2 helicase in the common synthesis dependent strand annealing pathway of homologous recombination in fission yeast. In this manuscript, the authors further delved into their functions and revealed that these proteins are required for proper DNA replication completion after hydroxyurea (HU) treatment. As observed in cells overexpressing Rrp1 and Rrp2, the overexpression of Srs2 resulted in chromosome instability and enhanced replication stress. Moreover, the authors demonstrated that the overproduction of Rrp1 Rrp2, and Srs2 differentially activated DNA damage and replication stress checkpoint responses. Although many of the data presented in this manuscript are clear, some specific points need to be addressed prior to publication.

Specific points
1) Fig. 1A, to claim that Srs2 is required for the full rescue of the rqh1∆ mutant’s HU sensitivity by rad57∆, the authors should rule out a possibility that the growth defect of the srs2∆rqh1∆rad57∆ mutant is due to the HU sensitivity caused by the srs2∆ mutation.

The way we understand the epistatic analysis between these three genes, both these statements are not exclusive, but are different ways of describing the same phenomenon.

Numerous studies have demonstrated that *srs2*∆ shows strong synthetic growth defect/is synthetic lethal with *rqh1*∆ mutant, demonstrating that these helicases act on alternative, at least partially independent pathways, and at least one is required for viability. This is suppressed by deleting *rhp55*+ or *rhp57*+ [3], showing that it is the accumulation of Rad57-dependent recombination intermediates that is toxic in the double *rqh1*∆*srs2*∆ mutant.

Here we focus on HU as one specific, source of DNA replication induced recombination intermediates. We cannot asses the sensitivity to HU of *rqh1*∆*srs2*∆ double mutant due to its huge growth defect, but by examining the sensitivity of the *rqh1*∆*srs2*∆*rad57*∆ mutant, we can uncover that both deletion of *srs2*+ and *rqh1*+ contribute. Thus, HU sensitivity of *rqh1*∆*srs2*∆*rad57*∆ mutant, which is greater than sensitivity of *rqh1*∆*rad57*∆ mutant, results from HU sensitivity of both *rqh1*∆ and *srs2*∆. This means that both helicases are involved in dealing with HR intermediates generated by HU treatment. In other words, Srs2 mediated alternative pathway for dealing with HU induced recombination intermediates allows the full rescue of *rqh1*∆ HU sensitivity by deletion of *rad57*+.

2) In the epistasis analyses shown in Fig. 1C and D, no additive effect was observed in the completion of replication after HU treatment when the srs2∆ mutation is combined with either rrp1∆ or rrp2∆. These results strongly indicate that Srs2 functions in the same pathway with Rrp1 and Rrp2 rather than acting independently. Therefore, I think that the author’s conclusion “the role of Srs2 in resuming replication after HU block may be independent of Rrp1 and Rrp2 (Line 206)” does not reflect the authors’ findings properly.

As we wrote in response to the Reviewer #1 we are very grateful for pointing out this obvious mistake we made. We wrote precisely the opposite of what we meant to write. We changed the wording and the relevant sentence now reads, line 255-258:

“The double mutants simultaneously devoid of *srs2+* and *rrp1+* or *rrp2+* did not show any further delay in the entry of chromosomes into the gel (Fig. 1B) suggesting that Srs2, Rrp1 and Rrp2 may have overlapping roles in the resolution of replication intermediates after HU block.”

3) In this manuscript, most of the experiments have been conducted using cells overexpressing Srs2, Rrp1 and Rrp2. Therefore, the author should confirm whether the expression level of those proteins is comparable to each other.

We now included in Fig. 2C the Western blot comparing Srs2, Rrp1 and Rrp2 protein levels in two different transformants. Srs2 seems more abundant, but the protein levels do not corelate directly with growth defect as overproduction of Rrp2 is more toxic than Rrp1 while the levels of both proteins are comparable.

4) Fig. 6A-C, although the authors claim that cell length was decreased to a similar degree in the chk1Δ and cds1Δ mutants when Srs2 was overexpressed, it appears to me that at least the cell length of the cds1Δ mutant is increased. Moreover, the cell length of both chk1Δ and cds1Δ mutants overexpressing Rrp2 was significantly shortened, though the chk1Δ mutant was longer than cds1Δ. Therefore, the author’s statement in Lines 364-369 may need to be reconsidered.

This point was also raised by Reviewer #3 so we realise we did not express ourselves clearly and seemed to imply that only one checkpoint pathway was activated by overproduction of studied proteins. We have now rewritten description of data presented in Fig. 6A-C, (lines 477-490), and redesigned complementing diagrams to emphasise that we identify the differences in the prevailing pathway activated in Srs2, Rrp1 and Rrp2 overproducing cells.

Minor points
5) Fig. 2D, the nucleus should be detected by DAPI staining. Why the overexpression of Srs2 is so heterogeneous among cells?

In *S. pombe* DAPI staining gives best results on fixed cells but compromises the quality of GFP signal.

Instead we examined localisation of overexpressed EGFP-Srs2-FLAG in a strain expressing Gar2-mCherry, a nucleolar histone chaperone, and included these data in new Fig. 3A. We confirmed microscopy data by ChIP analysis, presented in Fig. 3B.

The cultures examined are not synchronised, so cells are at different stages of cell cycle. Additionally, the expression system we employ does not allow to precisely regulate protein levels (differences in plasmid copy number in the cell, plasmid loss, long induction time for *nmt* promoter, etc), and for that reason we always examine at least several independent transformants.

6) Fig. 5C, the authors state that “DNA damage and replication checkpoint pathways may have redundant roles in augmenting the survival of cells overproducing Srs2 (Line321-324)”. This idea can be further confirmed by demonstrating that the Srs2 overexpression severely compromises cell growth of the chk1∆cds1∆ double mutant like in rad3∆.

Rad3 is an upstream kinase to both, Chk1 and Cds1, so we concluded that analysing *rad3*Δ mutant would suffice. We agree with the Reviewer that several experiments could be done to corroborate our data and it would be interesting to explore in greater detail checkpoint dependent and independent interactions of Srs2 with Chk1, Cds1 and Rad3 but we hope the Reviewer agrees that this falls outside the scope of this work.

**Reviewer #3:** In this manuscript Baranowska and coworkers assessed the role of DNA translocase Rrp1, Rrp2 and Srs2. These factors were previously described by this lab to regulate Rad51 recombinase and to promote SDSA in response to replication stress and/or DNA damage (DSB). According to their model, Rrp1, Rrp2 and Srs2 function together and counteract Rad51 activity in the Swi5/Sfr1 sub-pathway of HR, favoring gene conversion rather than crossing over. In this new study, they investigated the impact of the overexpression of Srs2 with Rrp1 and Rrp2. High level of Srs2 activates simultaneously DNA damage and replication stress response and Srs2 localizes in the nucleolus.

The introduction does not clearly present current knowledge of the subject and does not properly lay the groundwork for this study. Plus, assumptions underlying this study have not been clearly established which makes this work hard to follow. Overall, the take home message of this study is not clear to me.
The genetic experiments are well conducted, however it would need further study to support their conclusions, regarding checkpoint activation as an example. They made the interesting observation that Srs2 accumulates in the nucleolus, suggesting a function of Srs2 in rDNA maintenance. Although it was previously established (Yasuhira , 2009), they did not further investigate the function of Srs2 in rDNA stability.
The lab previously proposed that Rrp1, Rrp2 and Srs2 function together, but they showed here that these factors may function distinctly and at different loci (telomeres, centromeres, rDNA). This point was not discussed. How can they reconciliate their first observations with their new results?
I have the impression that only the surface has been touched without a clear conclusion to this study. I am afraid that I can’t support the publication of this study at this stage.

Major points:
-The introduction does not clearly present current knowledge of the subject and does not properly lay the groundwork for this study. The authors should cautiously present the current understanding of HR regulation in S. pombe. Second paragraph of the introduction looks like a list of results without connection between them. Functions of of Rqh1 in the HR pathway should be properly introduced as well with its links with Rrp1/2. Distinction between observations and assumptions should be properly presented. The current status of the entire knowledge of Srs2 in S. pombe should be precisely established. A graphical abstract may help to clarify the entire introduction.

We have rewritten parts of the Introduction and added in Fig. S1A a schematic diagram of homologous recombination pathways that are discussed, with marked position for Rqh1, Srs2, Rad57 and Rrp1-Rrp2. We hope that now the Reviewer will find our description satisfactory.

Figure 1:
Figure 1A: Survival curves should be presented as shown in their previous publication (NAR, 2013, Figure 6C).

It is true that we presented survival curves for the analysis of HU sensitivity of *rqh1*∆*rad57*∆*,* *rqh1*∆*rad57*∆*rrp1*∆ and *rqh1*∆*rad57*∆*rrp2*∆ mutants but the differences between these mutants were more readily apparent on spot test with dilutions of cultures plated on control and HU containing media, and that is the reason we showed only this assay in present manuscript.

We had, however, performed another type of experiment measuring survival of studied mutants after acute HU exposure (4 hrs at 12mM HU) relative to wild-type, but thought these data were redundant with spot test presented. We now include them as Fig. S1C, with description (lines 236-239).

To fully support the epistatic function of Srs2 and Rrp1/2, the author should also test rad57Δ rqh1Δ srs2Δ rrp1Δ and rrp2Δ quadruple mutants with HU.

It is very difficult to obtain stable multiple mutants devoid of genes important for homologous recombination, even if they are viable. On careful examination of spot test in Fig. S1B one can observe bigger, suppressor colonies appearing for triple *rqh1*∆*rad57*∆*rrp1*∆ (or /*rrp2*∆or /*srs2*∆) mutants. We attempted to construct two quadruple mutants required but they only formed small colonies after prolonged incubation so even though it suggested independent functions for Rrp1/2 and Srs2, we did not pursue this further. As we stated in our manuscript we turned to overexpression analysis in order to asses the interactions between these 3 studied proteins.

Figure 1C: For the WT, the authors claim that the doubling of intensity is due to the completion of replication, in this case number of cells should be double in the time course of the experiment (but not the mutants). Is that the case ? Accordingly, they should provide cell counts.

We did not determine cell numbers for the indicated time points. At the start point (As) all the strains were equally diluted to 5x10^6^ cells per mL and then at each time point equal volume of cultures was collected. Instead of measuring the density of cultures, we performed FACS analysis of DNA content for each strain used for PFGE experiment demonstrating that at the last time-point all mutants completed replication and accumulated in G2. We included these data in Fig. 1C and their description in the text (lines 258-261).

Figure 1D: “The double mutants simultaneously devoid of srs2+ and rrp1+ or
rrp2+ did not show any further delay in replication completion, suggesting the role of Srs2 in resuming replication after HU block may be independent of Rrp1 and Rrp2 (Fig. 1D).” I do not agree, it looks epistatic to me.
(minor point: the last paragraph lane 207-210 should be move to the next part lane 224)

We are sorry for this obvious mistake, pointed out by all Reviewers. We corrected the text and the relevant sentence now reads, line 255-258:

“The double mutants simultaneously devoid of *srs2+* and *rrp1+* or *rrp2+* did not show any further delay in the entry of chromosomes into the gel (Fig. 1B) suggesting that Srs2, Rrp1 and Rrp2 may have overlapping roles in the resolution of replication intermediates after HU block.”

Figure 2D:
EGFP-Srs2 localizes within the nucleolus. Does it affect rDNA stability? PFGE should be performed. 2D-gel analysis of the rDNA locus could have been assessed in srs2Δ mutant or when Srs2 is overexpressed to support their conclusions and also support their discussion (lane 425).

We thank the Reviewer for this suggestion. PFGE analysis of rDNA in *srs2*Δ has already been performed [4] and showed the changes in rDNA size upon loss of Srs2 helicase.

Here, we have now provided the direct evidence of Srs2 interaction with rDNA (by co-localisation of EGFP-Srs2 with Gar2-mCherry and by Srs2-EGFP ChIP-qPCR).

According to the Reviewer’s suggestion we performed 2D-gel analysis of the rDNA locus in wild-type and *srs2*∆ mutant, to focus on physiological role of Srs2 absence, and found that Srs2 is required for proper replication of rDNA arrays. Upon its loss, replication is severely altered on natural pause sites (rRFBs) that are located within rDNA arrays. These results are now presented in Fig. 3C-E with description in the text (line 354-364).
However, as stated above, the shortcoming of transformants overproducing EGFP-Srs2 is the variability of Srs2 levels in cell population, so multiple transformants need to be examined. 2DGE is a sophisticated and time-consuming technique, and due to the overall costs of sample preparation it would be very difficult for us to test multiple transformants by this approach.

We thus hope the Reviewer will find presented data satisfactory and sufficient to support conclusion of our paper.

Figure 5:
Checkpoint activation is consistent with Rad11 foci accumulation. Chk1-phosphorylation is visible by western blot. As a control, treatment with DNA genotoxic agent should be shown to visualize phosphorylation of Chk1-HA and to evaluate the intensity of the phosphor shift.

We included required control in Fig. 5D, we cropped the presented blot before because due to different culture conditions (YES medium) and loading it is not possible to use it to evaluate intensity of Chk1 phosphorylation. Nevertheless, we agree with the Reviewer that it is important to present this control in order to confirm the identity of respective protein bands.

Why the phosphorylation status of Cds1 has not been monitored? This should be done to support their claims (cf figure 6)

We agree that additional examination of the phosphorylation status of Cds1 could further confirm our finding that both Chk1 and Cds1 pathways are differentially activated in cells overproducing Srs2, Rrp1 and Rrp2, but we do not have a strain with tagged Cds1 nor established assay for Cds1 enzymatic activity. Obtaining these would delay publication of our work so we hope the Reviewer agrees that for the purpose of this manuscript data presented are sufficient to support its conclusions.

Figure 6:
“ Cell length was decreased to a similar degree in chk1Δ and cds1Δ mutants over-expressing srs2+ (Fig. 6A) suggesting that overproduction of Srs2 activated both DNA damage and replication stress response pathways.” According to the figure 6A, this is not the case. The effect of overexpression of Srs2 resembles to Rrp1, activating both checkpoints. I feel that these data do not support their conclusions. Failure in checkpoint activation in S-phase may lead to G2/M checkpoint activation.

Reviewer #2 also raised this point so it is obvious we failed to clearly present our findings. We have now rewritten description of data in Fig. 6A-C, (lines 477-490), and redesigned complementing diagrams to emphasise that even though both checkpoint pathways are activated in Srs2, Rrp1 and Rrp2 overproducing cells we identify the differences in the pathway that prevails.

1.	Dziadkowiec D, Kramarz K, Kanik K, Wiśniewski P, Carr AM. Involvement of Schizosaccharomyces pombe rrp1+ and rrp2 + in the Srs2- and Swi5/Sfr1-dependent pathway in response to DNA damage and replication inhibition. Nucleic Acids Res. 2013;41: 8196–8209. doi:10.1093/nar/gkt564

2.	Dziadkowiec D, Petters E, Dyjankiewicz A, Karpiński P, Garcia V, Watson AT, et al. The role of novel genes rrp1(+) and rrp2(+) in the repair of DNA damage in Schizosaccharomyces pombe. DNA Repair (Amst). 2009;8: 627–636. doi:10.1016/j.dnarep.2008.12.008

3.	Doe CL, Whitby MC. The involvement of Srs2 in post-replication repair and homologous recombination in fission yeast. Nucleic Acids Res. 2004;32: 1480–91. doi:10.1093/nar/gkh317

4.	Yasuhira S. Redundant roles of Srs2 helicase and replication checkpoint in survival and rDNA maintenance in Schizosaccharomyces pombe. Mol Genet Genomics. 2009;281: 497–509. doi:10.1007/s00438-009-0426-x
